# A public and patient consultation process as an aid to design a person‐centred randomized clinical trial

**DOI:** 10.1111/hex.13304

**Published:** 2021-07-05

**Authors:** Jacqueline Rix, Sharon Docherty, Alexander C. Breen, Philip Sewell, Jonathan Branney

**Affiliations:** ^1^ Department of Design and Engineering Faculty of Science and Technology Bournemouth University Poole UK; ^2^ Centre for Biomechanics Research AECC University College Bournemouth UK; ^3^ Department of Medical Science & Public Health Faculty of Health & Social Sciences Bournemouth University Poole UK; ^4^ Department of Nursing Science Faculty of Health & Social Sciences Bournemouth University Poole UK

**Keywords:** clinical trial, patient and public involvement, research collaboration, trial design

## Abstract

**Background:**

Involving patients and members of the public, together with researchers, in decisions about how studies are designed and conducted can create a study that is more person‐centred. The aim of this consultation process was to explore ways of designing a study which takes the person into consideration for the randomized clinical study entitled ‘Biomechanical Effects of Manual Therapy—A Feasibility Study’ using the novel approach of usability testing.

**Design:**

Patient and public volunteers were sought with experience of low back pain. Volunteers were invited to participate in usability testing (a physical walkthrough) of the proposed study method. This was followed by a discussion of areas where usability testing could not be used, such as recruitment strategies, continuity of participant care and dissemination of results. Resulting feedback was considered by the research team and alterations to the original study method were incorporated, provided the research questions could be answered and were practical within the resources available.

**Results:**

Additional recruitment strategies were proposed. Alterations to the study included reduction in study time burden; completion of study paperwork in a quieter location; continuity of participant care after the study; and methods of dissemination of overall study results to participants.

**Conclusion:**

The consultation process used the unique method of usability testing, together with a post‐usability discussion, and resulted in alterations to the future study which may facilitate making it more person‐centred.

**Patient and Public Contribution:**

Patients and public developed the future study design but did not participate in manuscript preparation.

## 1 BACKGROUND

Health care, in recent years, has seen a paradigm shift from medical autonomy and disease‐based care to a more person‐centred approach to care.^1^ The principles and concepts of person‐centeredness are now commonplace in national^2^, ^3^, ^4^ and global health care policies.^5^ There are also significant funding investments into providing tools aimed at health care professionals designed to improve person‐centred care,^6^, ^7^ as well as independent charities working towards improving care centred around the individual.^8^, ^9^ Health care research is following this paradigm shift, and significant efforts are being made to design research which takes the person into consideration.^10^, ^11^, ^12^

The term ‘person‐centred’ in health care is difficult to define, largely due to it being dependent on the care needs, circumstances and preferences of the individual receiving care.^13^ ‘Person‐centred’ is thought to differ from the term ‘patient‐centred’, as it focuses not only on the individual receiving health care (as a patient), but also on the person as a whole, living with their condition, in the context of their work, life and family.^14^ Care which is centred around the person has been demonstrated to be effective in a health care setting.^15^ Involving multidisciplinary teams, including patients, in clinical decision making as well as increased communication between patient and care provider appears to be more successful.^15^ However, the heterogeneity of the literature makes the effectiveness of this approach difficult to ascertain. This is partly due to the lack of a definitive definition of person‐centred care which results in significantly different study designs in the literature, but also due to a lack of a consistently utilized outcome measure with which to assess effectiveness.^15^

Typically, research studies have been designed by researchers with little or no input from the patients or members of the public.^10^, ^11^ Thus, studies tended to be researcher‐driven or researcher‐centred.^10^, ^12^ In recent years, there has been a move from researchers carrying out research ‘on’ or ‘to’ participants, to a more inclusive research design whereby it is carried out ‘with’ participants.^12^ Involving patients and members of the public, together with researchers, in decisions about how studies are designed and conducted can create a person‐centred study, echoing the changes in health care.^11^

Participation in research studies can be burdensome on participants. Therefore, when designing a study, the psychological, physical and financial burdens of participation should be recognized and minimized as much as possible.^16^ Considerations may include avoiding an overwhelming number of visits to the study site, or burdensome study requirements requiring a large time commitment from participants.^17^, ^18^ The design may also acknowledge that participants have busy lives and are juggling various work, life and family commitments.^19^ Research participants have highlighted the importance of good communication; for example, having the researcher clearly expresses that their participation is valued and ensuring continued care and support from researchers at the end of their participation.^20^, ^21^ In developing and designing a study that is based around the participant, these important aspects should be maximized.

It is important to understand the potential participant population.^11^ One of the ways to achieve this is to involve the people from that population and invite them to provide their input in building the study design and protocol.^17^, ^19^ There is some discussion in the literature regarding methodology for involving patients and members of the public in research.^22^, ^23^ INVOLVE^24^ suggests patient and public involvement may include a consultation, a collaboration or user‐led research. A consultation involves patients and the public to advise on either an aspect of the study or throughout the research study; collaboration involves the patients and the public as integral members of the research team; and user‐led allows people with the lived experience of the condition to take the lead in study direction and design.^25^ Involvement needs to be flexible to the needs of research studies and research methods, rather than a ridged token addition to a pre‐designed study.^22^

Literature suggests that simulations have been used to give patients and members of the public a chance to experience the research study method.^26^ This is not always possible, particularly if the aim is to contribute to the design of a future study, where the study design has not been finalized. Equally, there may be ethical considerations if the study involves potentially invasive investigations or treatment. For this reason, an alternative method of patient and public consultation may need to be considered, such as usability testing. Usability testing is extensively used in computer engineering fields. It was introduced by Lewis^27^ and later refined by Ericsson and Simon.^28^ The aim is to gain an understanding of users and identify the main problems associated with using a system.^29^ During the consultation, volunteers are encouraged to keep talking and focus on how they experience the system in their own words, with minimal intervention from the researcher.^29^ This differs from other usability tests, such as cognitive walkthroughs which are usually carried out by an analyst or engineer (fellow expert in the field), and not the end‐stage user. There is a paucity of literature relating to the use of a usability testing as an aid to designing clinical studies, as such this is a novel approach to a patient and public involvement consultation.

This patient and public involvement process utilized a targeted consultation process and involved patients and the public in one aspect of the study design,^25^ to assist in creating a more person‐centred study from a pre‐existing study method for the randomized clinical trial (RCT) entitled: Biomechanical Effects of Manual Therapy—A Feasibility Study. As this was a feasibility study, a targeted consultation process was used, rather than collaboration or user‐led involvement as a large group of volunteers could be recruited for maximum feedback on one aspect of the study design.

The resulting RCT will look at biomechanical changes associated with acute low back pain. As such, patients currently having treatment for low back pain and members of the public who have had experience of low back pain were invited to participate in usability testing of the proposed study method. This was followed by a post‐usability test discussion for areas of the method where usability testing could not be utilized.

## 2 METHOD

### 2.1 Ethics

This patient and public involvement was a consultation process and not considered research by the NHS.^30^ Following completion of the HRA NHS Review decision tool^31^ and under the advice of local ethics, ethical approval was not required.

### 2.2 Recruitment

Adult public and patient volunteers were sought with current or prior experience of low back pain. Volunteers were recruited via the university public and patient partnership, as well as an advertisement displayed in the reception of the university's private teaching clinic. Involvement was voluntary, and volunteers were not paid for their time. All interested volunteers were sent an email containing details of the consultation process including.

- The role of the volunteer in the consultation process. Volunteers were being recruited to assist in the design of a research study to make it as participant‐friendly as possible. Their experience of low back pain allowed volunteers to view the study design from the participant's standpoint, which placed them in a unique position to provide valuable feedback.
- What to expect on the day of the consultation process.
- Date and time the consultation processes were taking place. Two dates and time slots were available.

An additional date was arranged with two volunteers as they were unavailable for the proposed dates. No more than five volunteers per time slot, this was largely dictated by the need to minimize disruption in a busy clinic during opening hours. A total of nine interested volunteers responded to the advertising, all responders took part in the consultation process.

### 2.3 Consultation process

**Figure 1:**
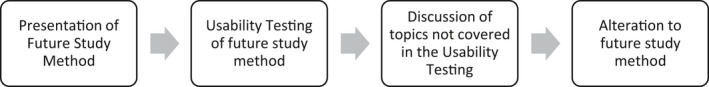
Outline of the consultation process aimed at exploring the most person‐centred way of carrying out the clinical study

Volunteers agreed to: voice recording of the consultation process; future contact for the purposes of discussion clarification; and named acknowledgement in future publications if they wished. The process followed that set out in Figure 1.

The aims and objectives of the future study, and how it would contribute to existing knowledge related to low back pain were outlined to the volunteers. This provided background information to enable a better understanding of the study. An outline of the proposed study method (Table 1) was handed out to support discussion between the researcher and volunteers.

**Table 1:** Outline summary of the future study method. The study is a two‐arm randomized clinical trial investigating the biomechanical effects of manual therapy

Timeline	Study Stage	Details of study stage	
	Recruitment	Recruitment carried out in private university teaching clinic; patient identified; patient eligibility established at the new patient examination.	
Day 1	Baseline measurements	Participant consented into study; back pain questionnaires; pre‐fluoroscopy form (pregnancy statement); fluoroscopy (moving video X‐rays)	
Day 2 to day 13	Intervention	Both groups receive a home management booklet.	
		Group 1: Five manual therapy appointments within two weeks	Group 2: No treatment appointments
Day 14	Follow‐up measurements	Back pain questionnaires; pre‐fluoroscopy form (pregnancy statement); fluoroscopy (moving video X‐ray)	
	Study completion	Signposting for further treatment once study is complete; dissemination of results of study	

The consultation process was carried out in two parts; all volunteers took part in both parts.

### 2.4 Usability testing

**Figure 2:**
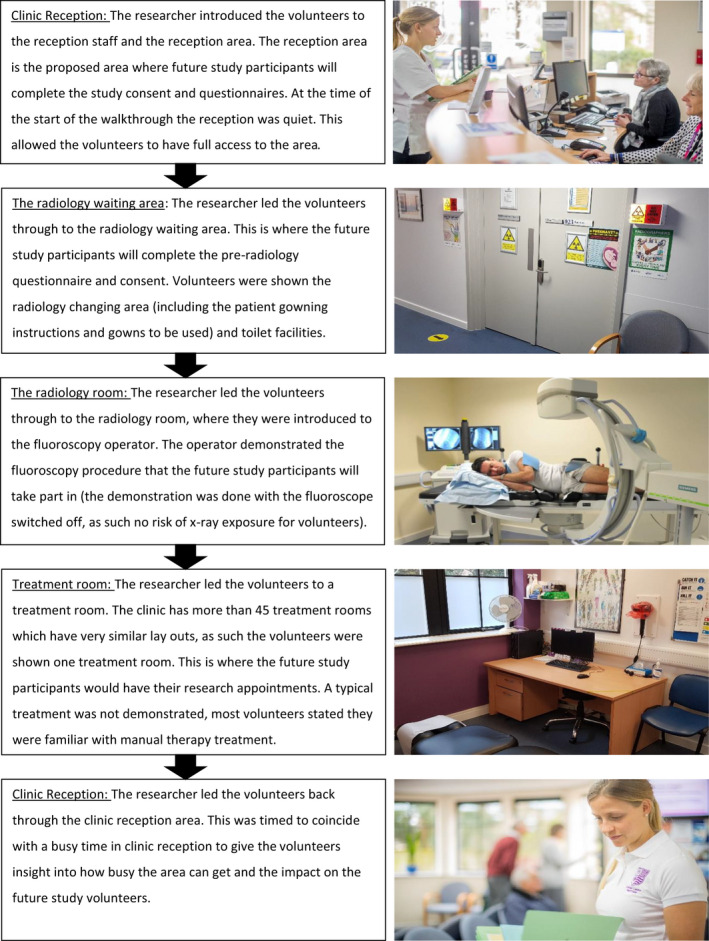
Flow chart of the usability testing, outlining each room of the clinic involved in the future study and what will be taking place in each room

Volunteers were walked through the physical environment of the clinic, and what would be expected of study participants in each of the study locations was described (Figure 2). Walking the volunteers through the physical environment linked the study expectations to the physical space in which it would take place. Stopping and exploring each room provided insight into the reaction of future participants to the study experience. Volunteers were encouraged to ‘think aloud’ in each room and respond to the activity description. They were also given a clipboard, paper and a pen to make additional notes.

### 2.5 Post‐usability test discussion

Following the usability testing, a discussion took place in a quiet environment. The researcher‐led discussion focused on areas of the study not addressed during the usability testing. The discussion was based on a semi‐structured focus group format to ensure all volunteer groups discussed similar topics.

### 2.6 The topics for discussion were as follows

- Recruitment strategies.
- Participants’ willingness to be randomized.
- Treatment schedules for both arms of the randomized clinical study.
- Continuity of patient care once the research study is complete.
- Dissemination of study results to participants.

Discussions lasted a maximum of 30 minutes. Any additional notes taken by the volunteers during the usability testing were collected. At the close, volunteers were thanked for their assistance.

### 2.7 Feedback

Feedback was collated by the researcher who carried out the consultation process and compiled into one document (Microsoft^®^ Word for Microsoft 365, USA). All researchers discussed the feedback from the consultation process and decided which areas of the study required alterations; whether any alterations may impact the research questions; and whether the alterations to the study were practical and achievable for the clinic layout and resources. Agreed alterations were made to the future study method to create a study which took the individual participants into consideration.

## 3 RESULTS

Three consultation processes took place, with a total of nine volunteers. There were five volunteers in the first while the second and third comprised of two volunteers each. One male and eight females took part in the process, with an age range of 24‐76 years of age. The ethnic group of all volunteers was white (British).

### 3.1 Usability testing recommendations

#### 3.1.1 Clinic reception

It was felt that the waiting room was very busy and noisy, and as such, other places for the filling out of forms and questionnaires were discussed. A treatment room was thought to be more comfortable for the participant, where it is quiet. Volunteers also felt it was awkward to complete questionnaires and forms while sitting in a chair with a clipboard. As the participants will be suffering from back pain, volunteers felt they may need a little space to move around if needed.

#### 3.1.2 The radiology waiting area

The radiology waiting area is smaller, less noisy and more private. This was considered by one volunteer group as an area where the consent process, questionnaires and pre‐fluoroscopy forms could be completed. The remaining two groups felt that a treatment room would be the best option.

#### 3.1.3 The radiology room

The volunteers enjoyed the fluoroscopy demonstration and felt that both the researchers present made them feel comfortable. The volunteers acknowledged that the room contained lots of ‘*scary looking complicated equipment*’, but the personal interaction with the researchers, and demonstration of the equipment made the process of a fluoroscopy less intimidating.

#### 3.1.4 The treatment room

As most of the volunteers have had treatment at the university teaching clinic before, it was acknowledged that all rooms are essentially the same. It would be preferable to get a treatment room close to the radiology suite for ease of getting to and from the fluoroscope.

### 3.2 Post‐usability test discussion

#### 3.2.1 Recruitment

Volunteers were interested in discussing additional recruitment strategies:

- Volunteers discussed the option of recruitment via general practitioner (GP) surgeries as a viable option.
- Private practice recruitment was discussed, it was felt that the clinicians may feel that paying patients are being taken away from them, and as such, the volunteers felt this may not be a viable option.
- Recruitment via hospitals was discussed; the researcher outlined that these patients may not fulfil the inclusion/ exclusion criteria of the future study.

Regarding the approach to potential participants for the study by the researcher, volunteers discussed that potential participants may like time to consider whether to take part in the study or may want someone else present in the room. The researcher informed volunteers that potential participants were given 24 hours to decide whether to take part in the study or not.

#### 3.2.2 Randomization

The researcher led a discussion on what randomization is, and the two groups of the clinical study. The researcher had concerns regarding willingness of participants to be randomized. The volunteers felt that the information sheet provided to potential future study participants was well written and explained the randomization process and what would happen to the participant in each group. As such, if potential participants did not want to be randomized, they will not join the study.

#### 3.2.3 Appointment schedules for both groups

An in‐depth discussion was had by the volunteers regarding the non‐manual therapy group. This group will receive a fluoroscopy at the first and last research visit, and a Home Management Booklet. One volunteer group discussed that the participants in this group may feel as if they are left on their own to cope and as such have a higher risk of dropout. As a result of the discussion, an additional appointment halfway through the research will be made with participants in the non‐manual therapy group (see Table 2). While another volunteer group seemed to pick up on my wording when explaining the two groups and gave feedback that I could be more encouraging and positive when discussing this study arm. Home management (advice and reassurance) is a recognized form of treatment for low back pain, but potentially participants may not view the booklet as that, and it may need to be discussed and explained to the participants. The researcher should try to use wording that evokes participant empowerment (Volunteer Quotes: ‘*You can control the progress of your back pain*’; ‘*you can control your own back pain*’*)*.

**Table 2:** Outline of original proposed appointment schedule and the alterations made following the consultation process for both research groups

Timeline (days)	Group 1: Manual therapy		Group 2: Non‐manual therapy	
	Appointment schedule before PPI	Appointment schedule after PPI	Appointment schedule before PPI	Appointment schedule after PPI
1	Both groups receive a Home Management Booklet			
	Baseline measurements (fluoroscopy and questionnaires)	Baseline measurements (fluoroscopy and questionnaires) and first manual therapy appointment	Baseline measurements (fluoroscopy and questionnaires)	Baseline measurements (fluoroscopy and questionnaires)
2‐13	Five manual therapy appointments	Three manual therapy appointments	No appointments	Appointment halfway through the study.
14	Follow‐up measurements (fluoroscopy and questionnaires)	Final manual therapy appointment and follow‐up measurements (fluoroscopy and questionnaires)	Follow‐up measurements (fluoroscopy and questionnaires)	Follow‐up measurements (fluoroscopy and questionnaires)

Regarding the manual therapy group, this group's participation includes a first research visit which includes fluoroscopy (study day 1); followed by five manual therapy appointments (study day 2‐13); and followed by the last research appointment which includes fluoroscopy (study day 14). One volunteer group suggested that when thinking about driving to and from appointments and research load on participants, this was a lot of appointments in 2 weeks. Could they be cut down? This was discussed at length between researchers and it was concluded that the first manual therapy treatment would take place at the first research visit (study day 1); followed by three manual therapy appointments (study day 2‐13) and the fifth manual therapy treatment would take place at the last research visit (study day 14), thus reducing the appointment total from seven to five appointments (see table 2).

#### 3.2.4 Continuity of care

Upon completion of the study, participants will be signposted back to the original clinician who completed the New Patient Appointment. The volunteers thought this was an excellent idea, it allows continuity of care for participants. Clinicians will also have access to all research documentation related to the participant, such as treatment notes, fluoroscopy images and completed questionnaires.

#### 3.2.5 Dissemination of results

Volunteers thought it was important to provide participants with a summary of the study results as they had a vested interest in the outcome of the study.

## 4 DISCUSSION

All volunteers provided feedback during the consultation process and were willing to enter discussions on trial improvements. As a result of the discussions that took place during the consultation process, several changes will be included in the design of the future trial including recruitment; location for questionnaire completion; the consent process; randomization; the appointment schedule burden; continued support of participants; continuity of care; and dissemination of results.

### 4.1 Recruitment

The current feasibility study proposes single‐site recruitment at a university teaching clinic. However, a future fully powered randomized control trial would need to recruit from a larger pool of volunteers to meet the required sample size. During the post‐usability test discussion, volunteers provided valuable thoughts on additional potential participant identification and recruitment sites. Recruitment from GP practices in the area, private practices (musculoskeletal health care providers) and hospitals were discussed. Each of these options would require further investigation as to the feasibility of using these additional Participant Identifying Centres, and a Participant Identifying Centre Agreement would need to be completed.^32^ While this is not an obstacle, it will require further resources and it is recommended that this should be considered at the proposal stage and not as an amendment or addition to an existing project.^33^

Recruitment at the university teaching clinic will take place at the New Patient Appointment. While the New Patient Appointment will be carried out by a student intern (final‐year chiropractic student), if the patient appears eligible for the study the researcher will then approach the patient. As means of introduction, they will give a brief outline of the study and hand out an information sheet. Involving the researcher in recruitment aids development of a trusting relationship with the researcher and opens lines of communication from the outset. All of this is thought to aid person‐centred recruitment.^20^, ^21^ It will also allow potential participants to ask questions related to the study from a researcher who is better versed in the study method. This facilitates open dialog between the researcher and the potential participant when discussing the option of joining the study.^21^ Shared decision making allows the researcher and potential participant to converse about the best course of care for the individual, which may or may not be the research study.^34^ As the decision to take part in any research study should not be taken lightly, the volunteers in this PPI process felt that potential participants may want to be given the opportunity to have an additional person in the room with them. This is mirrored in the literature where it is suggested that researchers should encourage potential participants to speak to their family members to aid the decision‐making process.^33^

Volunteers felt that potential participants should not have to decide at the New Patient Appointment as to whether they would like to join the study. This had been considered during the study design by the researchers as it is suggested in the HRA guidance for consent and participant information.^33^ All potential participants will be asked for permission to be contacted telephonically by the researcher after 24 hours. There is no fixed guidance on the amount of time a potential participant should be given^33^; however, the study has an inclusion criteria of patients suffering from acute non‐specific low back pain. Due to potential participants being in acute pain, it was thought that 24 hours would be sufficient time for the participant to consider taking part in the study while balanced with receiving care in a timeous manner. While the researcher will contact the potential participant in 24 hours, they may request further time to decide whether they would like to take part in the study.

### 4.2 Consent and baseline measurements

Once a study participant decides to take part, a baseline measurement appointment will be scheduled. During this appointment, the information sheet will be discussed and written informed consent will be completed in accordance with the HRA guidance.^33^ While the content of the information sheet; the consent form; and the questionnaires were subject to a separate stakeholder consultation process,^35^ the location for the consent process and completing questionnaires was discussed. A treatment room was thought to be best option for this activity due to the room being quieter and more private. It is vital that a future study participant understands fully what the study is for; what their involvement will be; the risks involved with taking part; and alternative treatment options, before signing an informed consent.^33^ It is suggested that an information sheet and consent form, together with a meeting with a research team member for an extended discussion, can improve understanding of the study.^36^ It would be difficult to have a private discussion in a busy waiting room, and as such, the suggestion of using a treatment room would be the best option. A treatment room would also give the participant the option of a chair and desk to complete the consent and study baseline questionnaires, as well as room to stand and walk around if needed. The volunteers felt that completing paperwork using a clipboard in a busy waiting area would be uncomfortable, and the option of sitting at a desk with a comfortable chair would be welcomed by participants. As participants will be in acute low back pain, it was felt the option of walking around during the appointment would also be welcomed. As majority of the volunteers had or have had episodes of acute low back pain, their experience provided invaluable feedback for the creation of an environment which takes participant comfort into consideration.

During the baseline measurement appointment, study participants will have a fluoroscopy investigation of their low back. The radiology suite does have a number of ‘*scary looking complicated*’ machines, as a clinician and researcher working with these machines daily, one forgets how intimidating they can appear.^35^ For the usability testing, the fluoroscopy was demonstrated and explained. The volunteers felt that this put them at ease with the equipment and as such recommended a brief explanation of the equipment for the study participants. This contributes towards fully informed consent, whereby it is vital that study participants understand what their involvement entails and potential risks.^37^ As such, the brief demonstration will not only contribute to putting the study participants at ease, but ensure they fully understand the investigation they are about to take part, supporting the notion that research should be carried out ‘with’ the participant and not ‘to’ the participant.^12^

### 4.3 Randomization

Following baseline measurements, the study participants will be randomized onto one of two groups. While the researcher had reservations about participants’ willingness to be randomized, the volunteers did not. Volunteers felt that all participants were given adequate detail in the study information sheet as to what the two groups involved. Participants not willing to be randomized would not take part in the study. The future study is a feasibility study, and as such, willingness to be randomized will be explored as part of the study and the proposed randomization process may be refined or altered following the outcome. Potential study participants who do not wish to take part will be asked whether they are willing to give a reason as to why. Information may give further insight into participants’ willingness to be randomized.

### 4.4 Appointment schedule

The volunteers were open to discussing the appointment schedules for both groups of the study. They felt that the non‐manual therapy group had a chance of ‘drop out’ as this group was only seen by the researchers for their investigations. The volunteers suggested an additional appointment halfway through the study would be helpful to allow the study participants to make contact with the researcher and gain reassurance and advice if needed. On‐going communication fosters a positive relationship and can be reassuring to study participants,^20^, ^21^ as such the appointment schedule for this group was altered for the study. Equally, the language used by the researcher may lead to potential dropout in the non‐manual therapy group. This highlighted the need to be more cognisant of wording used to describe the trial arms. It is suggested that participants who have a more positive interaction are more likely to view the study more positively.^20^

Regarding the manual therapy group, the volunteers felt that the research burden on the study participants was large as there could potentially be seven appointments in two weeks. The literature mirrors the concern of patients regarding overwhelming numbers of appointments or large research burdens on patients.^16^, ^17^ Five treatments in two weeks are recommended by treatment guidelines; however, as a result of the feedback from the volunteers, it was decided that the first treatment would be carried out in the same appointment after the first fluoroscopy, and the last treatment would be carried out in the same appointment before the last fluoroscopy; as such, the study participants would only have five research appointments in total, rather than the original seven. Although this would make the first and last appointments longer, participants who may be travelling a distance for the trial would ultimately save time as well as travel costs.

### 4.5 Continuity of care

Once a participant has completed the study, they will be signposted back to their original clinic intern (final‐year chiropractic student); thus, they would not have to start again with someone new. The unique experience of the volunteers of having been treated within the university teaching clinic highlighted the importance of continuity of care for the future study participants, which is consistent with the literature.^20^

### 4.6 Dissemination of results

The volunteers felt that if participants had given their time to be a part of the study, they should be informed of the study outcome, which is supported in the literature.^20^ As such, changes were made to the study consent form to include an additional optional tick box ‘*I am interested in the overall results of the research. I would like the overall results emailed to me upon completion of the research. I agree to my email address being used for this purpose*’.

Interestingly, during the usability testing, volunteers were focused on the physical rooms, although they were introduced to the receptionists and fluoroscope operators. There was very little feedback relating to the people who the future participants will be in contact with. One of the keys to developing a person‐centred study is communication and reassurance.^20^ While much of this will come from the researcher, the whole health care team is instrumental in providing this.

This usability test and discussion resulted in changes to the original study method with the aim of producing a more person‐centred study design. The method of this consultation process was unique in a health care study development setting. Many patient and public involvement processes encourage payment of volunteers for on‐going research collaboration, or expenses reimbursed for a ‘one off’ involvement.^38^ During recruitment for this consultation process, volunteers were informed that no payment would be provided, which is generally considered poor practice.^39^ However, a reward may be offered which is not necessarily financial, and as such, volunteers were provided with refreshments during the consultation process and asked whether they would like to be acknowledged in any resulting publications.^39^ Future studies should consider building in a public and patient involvement process into the proposal and budget calculations of a study. The method is most likely more time‐consuming than a cognitive walkthrough, which would use fellow experts in the field such as fellow clinicians or researchers. However, the benefits of using a participant representative population outweigh the time burden for researchers. There is a growing need for a wider range of voices to be heard in study development and research, such as Black, Asian and minority ethnic populations (BAME).^40^ This consultation process advertised for, and welcomed, all adults from any ethnic group. However, responses were only obtained from one ethnic group, which is generally considered a weakness as not all voices are heard. For this reason, future public involvement processes should aim to include under‐represented groups.

The original study method had already been viewed by the team of researchers; the volunteers were able to view the study through the eyes of a participant. This resulted in recommendations and changes to the study the research team had not considered. As such, this consultation process was invaluable in helping to create a more person‐centred study. It should be reiterated that the future study is a feasibility study, and as such, the alterations suggested by the volunteers can be implemented, reflected upon and possibly refined before the final study protocol is established.

## 5 LIMITATIONS

The age range of the volunteers (24‐76 years of age) is slightly older than the age range of the future study which is 18‐65 years of age. Gender representation within the consultation group was skewed as only one of the volunteers was male, the remaining volunteers were female. It is proposed that a gender gap in research participation, especially when voluntary (unpaid), is influenced by gender roles, responsibilities and gender‐specific decision‐making processes.^41^ Females are significantly more likely to volunteer for research based on general altruistic considerations.^41^ The significant gender gap evident in this consultation process was not thought to influence the outcome of the process.

It is unknown whether the lack of reimbursement influenced who volunteered or the outcome of the consultation. Furthermore, the lack of ethnic diversity on the outcome of the process cannot be discounted.

## 6 CONCLUSION

The consultation process used the unique method of usability testing, together with a post‐usability discussion to aid the design of a more person‐centred study. The process resulted in alterations to the future study, including participant recruitment, location of study paperwork completion, study appointment schedule, continuity of care and informing the participants of the study outcome. It is hoped that these alterations may facilitate making the future study as person‐centred as possible.

## CONFLICT OF INTEREST

The authors declare that they have no conflict of interest.

## AUTHOR CONTRIBUTIONS

All authors have made a substantial contribution to conception and design. JR carried out the consultation process and collation of feedback. All authors were involved in the discussions of alteration to the original study design. JR was involved in drafting the manuscript, and all authors were involved in revising the manuscript. All authors have given final approval of the version to be published. All authors agree to be accountable for all aspects of this work.

## Data Availability

Data sharing not applicable to this article as no datasets were generated or analysed during the current study.
